# Harnessing the Medicinal Potential of Enset (*Ensete ventricosum*) in Ethiopian Traditional Medicine: A Synthesis of Current Knowledge

**DOI:** 10.1002/fsn3.71169

**Published:** 2025-11-25

**Authors:** Mitiku Muanenda, Workineh Mengesha Fereja, Wadzani Palnam Dauda

**Affiliations:** ^1^ Department of Horticulture College of Agriculture and Natural Resources, Dilla University Dilla Ethiopia; ^2^ Department of Chemistry College of Natural and Computational Sciences and Energy & Environment Research Center Dilla University Ethiopia; ^3^ Department of Agronomy (Crop Science Unit) Federal University Gashua Federal Nigeria

**Keywords:** aliment treated, challenges, ethnic groups, medicinal enset landraces, medicinal uses, scientific justification

## Abstract

Enset (Ensete ventricosum) plays an important role in Ethiopian traditional medicine. A tremendous number of enset landraces are traditionally used for medicinal purposes in almost all enset‐growing areas of the country. However, research on the medicinal applications of enset has so far been fragmented, focusing mainly on specific areas or ethnic groups, and no comprehensive national‐level synthesis exists. The objective of this review was to summarize and organize the traditional medicinal use of enset in Ethiopia. Research reports were searched on platforms such as Web of Science, Google Scholar, Scopus, AGRIS, and PubMed. Relevant research reports were selected, reviewed, and the required data were extracted and organized using Microsoft Excel 2016. Available research reports indicated that more than 600 enset landraces have been documented from 15 enset growing areas, of which about 100 (16.7%) are used in traditional medicine. Among these areas, the Gurage Zone and Amaro Special District recorded the highest number of medicinal landraces (12 each). Different enset parts, such as corm, pseudostem, and leaves, and enset products, including boiled corm, bulla, kocho, and sap from pseudostem, are used for medicinal purposes for both humans and livestock. Of these, corm and bulla are the most frequently utilized (89%). The most commonly treated ailments include bone fractures (29%), back pain (23%), joint displacement (21%), and placenta discharge (19%). Scientific investigations, including nutritional, molecular, and phytochemical studies, suggest that enset has significant medicinal potential. It is rich in arginine, calcium, zinc, and iron, and exhibits antioxidants, antitumor, antibacterial, antifungal, and nematicidal properties. Overall, medicinal enset landraces play a crucial role in traditional healthcare systems; yet they are increasingly threatened by various factors. Therefore, sustainable utilization, conservation, and further scientific validation of these landraces should be considered priority areas.

## Graphical abstract

This study provides the first national‐level synthesis of medical Enset (*Ensete ventricosum*) in Ethiopia. Over 600 Enset landraces were identified, with 16.7% documented from 15 major Enset growing areas, where corn and bulla are the primary medical parts used to treat bone and back ailments. The traditional medicinal uses of Enset are scientifically supported by its rich nutritional and phytochemical composition. Therefore, sustainable conservation and scientific validation of medicinal Enset are essential to realize its potential future therapeutic applications.

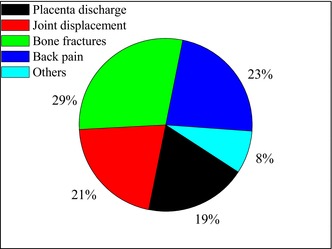

## 1 Introduction

Enset ( *Ensete ventricosum* ), which belongs to the order *Scitamineae*, the family *Musaceae*, and the genus *Ensete* (Simpson 2006), is the only species of this genus that is cultivated and consumed as a crop (Brandt et al. 1997). *Ensete ventricosum* is commonly named “Enset” or is sometimes called the “false banana” because it looks like the banana plant but does not produce fruit (Dejene 2024). In Ethiopia, it is widely known as Enset (in Amharic, the official language of the country) (Dejene 2024). The *Ensete ventricosum* was first published in the Kew Bulletin in 1947 and is a poorly characterized but resilient starch staple (Borrell et al. 2020; Paul et al. 2019). It is a giant herbaceous monocotyledonous plant with an adventitious root system and an underground stem structure known as corm (Negassa et al. 2025). The aboveground parts consist of a pseudostem formed from overlapping leaf sheaths, large leaves, and an inflorescence (Hedberg et al. 1997). *Ensete ventricosum* is distributed across Africa and Southern Asia (Kress et al. 2001), occurring widely in western, central, eastern, and southern Africa (Jones 2000). Despite its broad distribution, enset has been domesticated only in Ethiopia, where its cultivation as a staple food crop is restricted to the southwestern highlands, predominantly among communities speaking Semitic, Kushitic, and Omotic languages (Blench 2007). Ethiopia is therefore recognized as both the center of origin and the center of diversity for enset (Bekele 2018).

Enset is a staple food for nearly one‐fifth (approximately 20 million) of Ethiopia's population, particularly in the center, southern, and southwestern parts of the country (Negassa et al. 2025; Yemata 2020). The main food products derived from enset include kocho (a fermented product from the corm and pseudostem), bulla (a dehydrated starch extracted from the pseudostem and corm), and amicho (boiled corm) (Borrell et al. 2020). Enset is a multipurpose crop, and it has a vast role other than food value (Dilebo 2025a). Beyond its role as a food crop, enset is considered multipurpose, with traditional medicinal uses among its most valuable functions in the local community (Negassa et al. 2025).

The remarkable diversity of Enset landraces in Ethiopia has been shaped by centuries of evolutionary processes influenced by environmental variability, domestication, and cultural practices guided by indigenous knowledge and traditions (Muluken et al. 2024; Olango et al. 2014). A significant number of enset landraces are used in traditional medicine across nearly all enset‐growing areas of the country (Jarso et al. 2023; Gebre and Lemma 2019; Brihanu and Zerihun 2015; Tamrat et al. 2020; Assefa and Fitamo 2016; Mengesha et al. 2022). Traditional medicinal applications of enset include treatment for bone fractures, joint displacement, tonsillitis, abdominal pain, cough, wounds, postpartum recovery, placenta discharge, back pain, skin scabies, diarrhea, and birth control (Brandt et al. 1997; Negassa et al. 2025; Olango et al. 2014). Enset is widely regarded as an essential medicinal plant in enset‐growing areas, with consistent recognition across diverse ethnic groups. In some enset‐growing areas, enset is even ranked the most important medicinal plant compared to other species (Tefera and Kim 2019).

Scientific studies provided partial justification for these traditional uses. Nutritional and phytochemical analyses reveal that enset contains compounds and nutrients with potential medicinal roles (Bedada 2025). There is also a report on the morphological and molecular characterization of enset landraces, which are cultivated for traditional medicinal value (Hölscher and Schneider 1998; Tamrat et al. 2020). However, research on the medicinal use of enset remains fragmented, with studies often limited to specific geographic areas and ethnic groups. This fragmentation leaves gaps in understanding the broader national context, including the similarities and differences in the medicinal applications of enset across Ethiopia. Key questions remain regarding the extent of scientific evidence supporting its traditional uses, the distinctiveness of medicine landraces, and the challenges facing the preservation and sustainable use of enset for medicinal purposes. Therefore, this review aims to compile, summarize, and critically assess the existing fragmented research on the medicinal use of enset in Ethiopia, with the goal of providing a comprehensive national‐level perspective.

## 2 Methodology

### 2.1 Literature Search

A rigorous and systematic literature search was conducted across multiple electronic databases, including Web of Science, Google Scholar, Scopus, AGRIS, and PubMed, to ensure comprehensive coverage of relevant studies. The search strategy was meticulously crafted using a combination of pertinent keywords and Boolean operators to capture a broad spectrum of literature pertaining to enset's traditional medicinal use in Ethiopia. Keywords such as “enset,” “traditional medicine,” “ethnobotany,” and “Ethiopia” were utilized in various combinations to optimize search sensitivity. The search was not limited by publication date or language to encompass all potentially eligible studies and ensure inclusivity.

### 2.2 Study Selection Criteria

A set of predefined inclusion and exclusion criteria was established to guide the selection of studies for inclusion in the review. Eligible studies included original research articles, reviews, and gray literature documenting the traditional medicinal use of enset in Ethiopia. Specifically, studies reporting on enset landraces, parts utilized (e.g., corm, pseudostem, leaf), types of ailments treated, and associated traditional practices were considered. Additionally, articles providing information on nutritional, molecular, or phytochemical analyses relevant to Enset's medicinal properties were included. To maintain the integrity of the review process, studies not pertinent to enset's medicinal use or conducted outside the geographical confines of Ethiopia were excluded. Moreover, duplicates, conference abstracts, and articles lacking full‐text availability were also excluded from consideration.

### 2.3 Study Selection Process

The study selection process was conducted with utmost care and adherence to a meticulous two‐stage screening procedure, overseen by two independent reviewers. Initially, a comprehensive examination of titles and abstracts of retrieved records was undertaken to identify potentially relevant studies. Each record was meticulously scrutinized to ascertain its alignment with the predefined inclusion criteria. The reviewers applied stringent criteria to ensure that only studies directly pertinent to the traditional medicinal use of enset in Ethiopia were considered for further evaluation.

Following the initial screening phase, full‐text articles of the selected studies were retrieved and subjected to a thorough evaluation against the inclusion criteria. Each full‐text article underwent a detailed examination to determine its suitability for inclusion in the review. The reviewers meticulously assessed the content of each article, paying particular attention to the reported enset landraces, parts utilized, types of ailments treated, and associated traditional practices. Any discrepancies or disagreements between the reviewers during the selection process were meticulously documented and promptly addressed. To maintain the integrity and reliability of the study selection process, any instances of discordance or uncertainty between reviewers were resolved through thorough discussion and consultation. In cases where consensus could not be reached, a third reviewer was consulted to provide additional insights and facilitate resolution. Through this collaborative approach, consensus was reached on the inclusion of studies that met the predefined criteria, ensuring accuracy and consistency in study selection. Overall, the study selection process was characterized by a rigorous and systematic approach, aimed at identifying and including studies that provided robust and relevant evidence on the traditional medicinal use of enset in Ethiopia. The application of stringent criteria and the resolution of any discrepancies through consensus‐building mechanisms ensured the integrity and reliability of the selected studies, thereby enhancing the credibility and validity of the review findings.

### 2.4 Data Extraction and Analysis

In the data extraction phase, a systematic and structured approach was employed to capture relevant information from the included studies. A standardized data extraction form was meticulously designed to ensure consistency and comprehensiveness in gathering data across various dimensions of enset's traditional medicinal use. This form was tailored to capture key elements of each study, facilitating a detailed analysis of enset's medicinal properties and associated practices. The extracted data encompassed a range of essential parameters, including details on enset landraces, parts utilized in traditional medicine, types of ailments treated, and the specific traditional practices associated with enset‐based remedies. For each included study, information on the documented enset landraces, such as their geographical origin and morphological characteristics, was meticulously recorded to provide insights into the diversity and distribution of enset varieties used for medicinal purposes.

Furthermore, details on the specific Enset parts utilized in traditional medicine, such as the corm, pseudostem, and leaf, were carefully documented to elucidate the breadth and scope of Enset‐based remedies. This included information on the preparation methods and administration routes of enset‐derived products, such as boiled corm, bulla, kocho, and sap. In addition to documenting the types of ailments treated using Enset‐based remedies, the data extraction process also captured information on the associated traditional practices and cultural beliefs surrounding enset's medicinal use. This included insights into the traditional healing rituals, ceremonies, and indigenous knowledge systems that underpin the therapeutic efficacy of Enset in Ethiopian traditional medicine. Moreover, where available, data on nutritional, molecular, or phytochemical analyses relevant to enset's medicinal properties were extracted and recorded. This included information on the nutritional composition of enset‐derived products and any bioactive compounds identified through scientific analysis, such as antioxidants, antimicrobial agents, or anti‐inflammatory compounds.

The organized dataset facilitated subsequent analysis and synthesis of findings using both quantitative and qualitative approaches. Quantitative analysis involved summarizing and quantifying the frequency and prevalence of enset's traditional medicinal use across different studies and populations. Meanwhile, qualitative analysis involved synthesizing and interpreting the extracted data to identify recurring themes, patterns, and cultural insights related to enset's medicinal properties and traditional practices.

### 2.5 Quality Assessment

A comprehensive quality assessment of included studies was conducted to evaluate their methodological rigor and reliability. Various tools and checklists tailored to different study designs were employed for quality appraisal, ensuring a systematic and standardized approach. Studies demonstrating robust methodology and adherence to rigorous research practices were accorded greater weight in the analysis to enhance the validity and reliability of the synthesized evidence.

### 2.6 Sensitivity Analysis and Publication Bias Assessment

Sensitivity analysis was performed to assess the robustness of the findings by exploring the impact of excluding studies with methodological limitations or a high risk of bias. Furthermore, publication bias was assessed using funnel plots and statistical tests to detect asymmetry in the distribution of study outcomes. Addressing potential biases and uncertainties in the evidence base helped ensure the credibility and validity of the review findings.

## 3 Result and Discussion

### 3.1 Botanical and Taxonomic Description of *Ensete ventricosum*

*Ensete ventricosum* (Welw.) Cheesman is a giant, herbaceous monocotyledonous perennial plant. Its below‐ground structure consists of a massive, true corm (a swollen underground stem) and an adventitious root system. The above‐ground parts form a robust pseudostem, composed of tightly overlapping leaf sheaths, which can reach several meters in height. The plant produces large, oblong‐shaped leaves with a prominent midrib, arranged in a spiral. The inflorescence is a terminal, large, pendulous spike, and unlike its cultivated relatives in the genus *Musa*, *Ensete ventricosum* does not produce edible fruits, and the plant is monocarpic, dying after flowering and fruiting (Simpson 2006; Borrell et al. 2020).

### 3.2 Distribution of Medicinal Enset Landraces Across Enset‐Growing Regions

**FIGURE 1:**
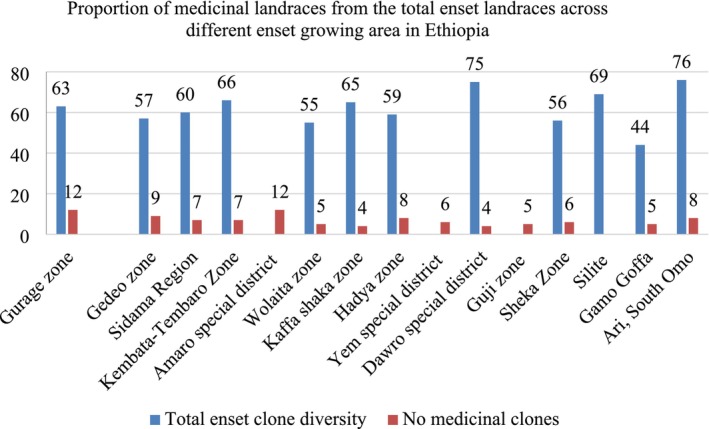
Proportion of medicinal landraces from the total enset landraces diversity across the enset growing area in Ethiopia.

The systematic review revealed a diverse distribution of medicinal enset landraces across various enset‐growing regions in Ethiopia. More than 600 enset landraces were documented, from 15 distinct enset‐growing areas, of which approximately 100 (16.7%) were reported to have medicinal applications (Tables 1 and 2). The distribution of medicinal enset landraces varied across different enset‐growing regions, with certain areas exhibiting a higher concentration of documented landraces. The Gurage Zone and Amaro Special District reported the highest numbers, with 12 medicinal landraces each. Substantial numbers of medicinal landraces were also recorded in other areas, such as the Gedeo Zone, Hadya Zone, and Sidam Region, while fewer medicinal landraces were documented in areas such as the Dawro Special District and the Guji Zone (Figure 1).

**TABLE 1:** Medicinal enset landraces diversity in the major enset growing area of Ethiopia.

No.	List of landraces	No. medicinal landraces	District/zone/ethnic group	References
1	Astara, Guare, Qibnar, Mymote, Lemmat, Charkima, Dere, Kibinar, Dare, Sinniwo, Agade, and Woret.	12	Gurage Zone	Nida (1996); Abdella et al. (2017). Jarso et al. (2023); Nuraga et al. (2020)
2	Qarase, Medalacho, Qorqoro, Shana, Gecha, Genta, Astaranechi, Astarakeyi, and Kake	9	Gedeo Zone	Adem and Kibatu (2020); Brihanu and Zerihun (2015)
3	Ado, Gentichcho, Midashsho, Gediwocho, Kitichcho, Asikala, and Chachcho	7	Sidama Region	Assefa and Fitamo (2016); Egziabher et al. (2020)
4	“Geshera,” “Tesa,” “Qeqele,” “Welagela,” “Cherqewa,” Qekile, and Gishiro	7	Kembata‐Tembaro Zone	Ayenew et al. (2016); Mulachew (2015)
5	Tameto, Kafile, Zarigula, Cicirika, Wujhaqa, Gaje, Sitete, Bazaze, Comale, Canga, Jolola, and Kafile	12	Amaro Special District	Gebre and Lemma (2019)
6	Agino, Gefetanuwa, Maziya, Halla, and Lochingia	5	Wolaita Zone	Olango et al. (2014)
7	Choro, Tayo, Shasi Wagi (Wagi Beli), and Ariko	4	Kaffa Shaka Zone	Tsehaye and Kebebew (2006)
8	Astara, Kiniwara, Gishra, Agede, Hywona, Mekelwesa, Kombotra, and Oniya	8	Hadya Zone	Tamrat et al. (2020)
9	Gariye, Deya, Karona, Kinkisar, Anchiro, and Asu	6	Yem Special District	Tamrat et al. (2020)
10	Arke, Tsela, Lochingia, and Meze	4	Dawro Special District	Tamrat et al. (2020)
11	Astaraa, Birraa, Kakkee, Qararsee, and Qoomaa	5	Guji Zone	Mengesha et al. (2022)
12	Shuri, Maacaa‐dami, Shuuri, and Noobo		Sheka Zone	Semman et al. (2019); Robi et al. (2024)

**TABLE 2:** Proportion of medicinal enset landraces compared to the total enset landrace diversity.

District/zone/ethnic group	Total enset landrace diversity	No. medicinal landraces	% Medicinal landraces	References
Gurage Zone	63	12	19.05	Yemataw et al. 2016; Nida (1996); Jarso et al. (2023); Nuraga et al. (2020).
Gedeo Zone	57	9	15.79	Adem and Kibatu (2020); Brihanu and Zerihun (2015)
Sidama Region	60	7	11.67	Tsegaye (2002); Assefa and Fitamo (2016); Egziabher et al. (2020).
Kembata‐Tembaro Zone	66	7	10.61	Yemataw et al. (2016); Mulachew (2015).
Amaro Special District	78	12		Gebre and Lemma (2019)
Wolaita Zone	55	5	9.09	Tsegaye (2002); Olango et al. (2014)
Kaffa Shaka Zone	65	4	6.15	Negash (2007); Tsehaye and Kebebew (2006)
Hadya Zone	59	8	13.56	Tsegaye (2002); Tamrat et al. (2020)
Yem Special District	59	6	—	Tamrat et al. (2020).
Dawro Special District	75	4	5.33	Yemataw et al. 2016; Tamrat et al. (2020)
Guji Zone	34	5	—	Mengesha et al. (2022)
Sheka Zone	65	—	—	Semman et al. (2019); Robi et al. (2024)
Silite Zone	69–72	9	—	Yemataw et al. (2016)
Gamo Goffa	34–44	—	—	Alemu and Sandford (1991)
Ari, South Omo	24–76	4		Shigeta (1992)

Furthermore, the specific documentation of medicinal landraces in various districts and zones provided valuable insights into localized medicinal practices and preferences. Each documented landrace exhibited unique attributes, including morphological characteristics and chemical composition, which likely influenced its selection for specific ailments and treatment practices. Overall, the findings highlight the widespread utilization of enset landraces for traditional medicinal purposes across diverse regions of Ethiopia. The findings underscore the role of environmental and cultural factors in shaping traditional medicinal practices across Ethiopia. The diversity and distribution of medicinal enset landraces highlight the depth of indigenous knowledge and the potential of enset as a culturally significant medicinal resource. The observed patterns further emphasize the need for further comprehensive documentation and preservation of medicinal landraces at a national level (Table 1).

### 3.3 Traditional Medicinal Use of Enset

**FIGURE 2:**
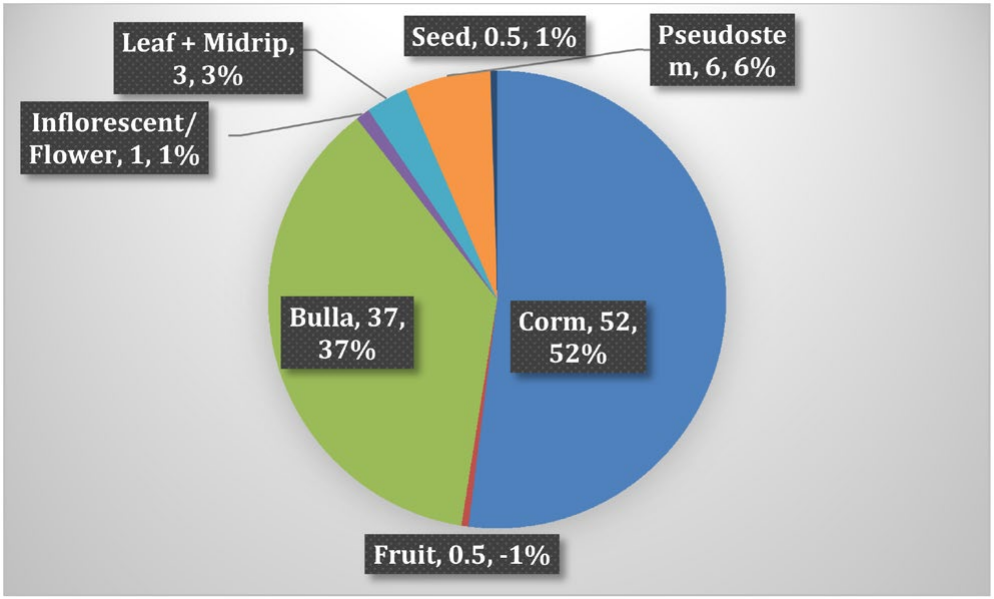
Proportion (%) of enset (*Ensete ventricosum*) parts used in medicinal practices.

Enset, often referred to as the “false banana,” holds a significant role in traditional medicine across enset‐growing regions of Ethiopia. Local farming communities have long recognized enset as a preferred medicinal commodity, recognizing its efficacy in treating a wide range of ailments. The systematic review of available reported research revealed widespread traditional medicinal uses of enset across all enset‐growing areas, as summarized in Table 3. The review identified multiple enset parts and products, including the corm, pseudostem, and leaf, as well as derived products such as boiled corm (amicho), bulla (dehydrated starch), kocho (enset leaf ash), and sequence water (sap extracted from pseudostem and leaf) that are utilized for medicinal purposes for both humans and livestock. Notably, the review highlighted that the corm and bulla were the most widely used parts of enset, accounting for approximately 89% of reported medicinal uses, as illustrated in Figure 2.

**TABLE 3:** Categories of medicinal landraces based on their use for different health alignments and the part used.

No of landraces	Local/vernacular names of enset landraces	Used part	Major medicinal uses of the landraces	Administrative zone/special district
16	Kibnar (G), Guarye (G), Dere (G), Astara (G, H, Ge), Kiniwara (H), Gishra (H), Agede (H), Gishiro/Gishra (KT), Tesa (KT), Chekiwa (KT), Sebera (KT), Arke (D), Tsela (D), Gariye (Y), Deya (Y), Nipho (Ge)	Corm and Bulla	Treatment of back pain, bone fractures, and joint displacement,	Gurage, Hadya, Gedeo, Yem Special District, Kabata Tembaro
5	Sinwot (G), Chehuyet (G), Terye (G), Hywona (H), Karona (Y)	Root	Expulsion of thorn and drainage abscess from a tissue	Gurage, hadya, Yem Special District
6	Bishaeset (G), Mekelwesa (H), Kiklenech (KT), Kikbgglekey (KT), Lochingia (D), Kinkisar (Y)	Boiled corm	Discharge of placenta following birth or abortion	Gurage, Hadya, Kembata‐Tembaro, Dawro, Yem
2	Atshakit (G), Kombotra (H)	—	Treatment of back injury	Gurage, Hadya
4	Oniya (H, KT), Beleka	—	Treatment of skin itching and Diarrhea	Hadya, Kembata‐Tembaro
	Kerese (Ge), Denkinet (G)	Squeezed water, fruit, boiled corm	Abdominal pain, malaria, hepatitis, and Eba and Aye disease	
3	Meze (D), Anchiro (Y), Oniya (H, KT)	—	Treatment of diarrhea	Dawro, Yem, Hadya, Kembata‐Tembaro
1	Agene	—	Treatment of coughing	Kembata‐Tembaro
1	Asu (Y)	—	For sewing a wound	
1	Qekile (KT)	Oiled corm	Placenta discharge	

*Note:* The letter in the bracket shows the location (Administrative zone/special district) of the landraces.

Abbreviations: D, Dawro; G, Gurage; Ge, Gedeo; H, Hadya; KT, Kembat‐Tembaro; Y, Yem Special District.

Analysis of the number of enset landraces utilized for medicinal purposes revealed that a total of 16 landraces across enset‐growing areas were documented to utilize their corm or bulla for medicinal uses. This indicates a significant proportion of Enset landraces contributing to traditional medicinal practices, with a notable preference for utilizing the corm or bulla. Porridge made from bulla, extracts from the pseudostem and leaf, boiled corm (Amicho), and highly fermented “kocho” were identified as the major enset‐derived products used in traditional medicine. Overall, the findings of this review emphasize the widespread utilization of enset in traditional medicine across Ethiopia's enset‐growing regions. The diversity of Enset parts and products utilized for medicinal purposes underscores the rich traditional knowledge surrounding Enset's therapeutic properties. These results provide valuable insights into the importance of Enset in indigenous healthcare systems and highlight the need for further research to explore its potential applications in modern medicine.

**FIGURE 3:**
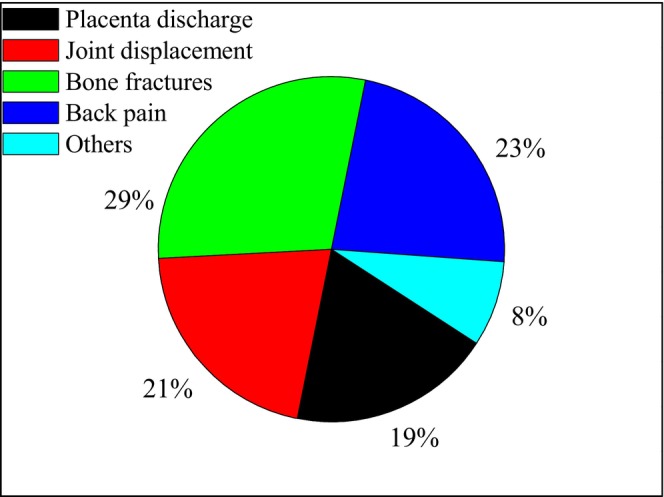
The percent (%) of the major ailments treated using *Ensete ventricosum*.

From many traditional medical practices, bone fractures, joint displacement, tonsillitis, abdominal pain, cough, wound, strengthening women after delivery, placenta discharge, restoring damaged parts of the body, back pain, preventing skin scabies, amoebiasis, and cramp are commonly reported (Table 3). Among these, the most frequently reported treated ailments are bone fractures (29%), back pain (23%), joint displacement (21%), and placenta discharge (19%) (Figure 3). On the other hand, the corm and bulla are dominantly used parts to treat back pain, bone fractures, and joint displacement.

### 3.4 Enset as a Potential Dietary Source of Essential Micronutrients

In a comparison with regionally important tuber and cereal crops, enset contains notably high concentrations of iron and zinc (Tamrat et al. 2020). This is particularly relevant in Ethiopia, where iron and zinc deficiencies are widespread. Anemia, primarily caused by iron deficiency, affects 56% of children and 24% of adult women in the country (Grebmer et al. 2018). This emphasizes the importance of enset as a potential dietary source of iron. Iron concentrations have been reported as higher in the enset pseudostem compared to corm (Heuzé et al. 2017; Tamrat et al. 2020). Similarly, zinc deficiency is a major dietary concern in Ethiopia (Gebru et al. 2018). Evidence shows that enset is an important source of zinc, with the corm containing higher concentrations than the pseudostem (Tamrat et al. 2020). These findings underscore the nutritional as well as medicinal value of enset in improving public health outcomes in Ethiopia (Dilebo 2025a).

### 3.5 Scientific Justification of the Medicinal Value of Enset

The nutritional, molecular, and phytochemical analyses of enset revealed that there is possible potential for enset to be used as a medicinal remedy (Table 4). Some of the supporting scientific studies for the traditional medicinal uses of enset include (1) phenylphenalenone content of enset, reported to have antitumor, antibacterial, nematicidal activity (Hölscher and Schneider 1998); (2) high calcium levels, particularly in corm, reported to have significant value for bone strength, growth and repair (Bizuneh 1984; Tamrat et al. 2020); (3) high arginine content, linked to collagen formation, tissue repair, and wound healing through its role in proline synthesis and as a precursor of nitric oxide (Tamrat et al. 2020); (4) iron and zinc richness, which are key micronutrients and their deficiencies are associated with major health problems such as anemia and impaired immunity (Tamrat et al. 2020); and (5) strong antioxidant capacity, with the corm showing a particularly strong ferric reducing antioxidant power (FRAP), indicating its ability to neutralize reactive oxygen species (Bedada 2025; Forsido et al. 2013).

**TABLE 4:** The major identified phytochemicals from enset, which are claimed to be of medicinal importance.

Name of phytochemical	Its medicinal use	References
Phenylphenalenone	Anticancer, antibacterial, and nematicidal properties	Hölscher and Schneider (1998).
Total phenol content	Antioxidant	Forsido et al. (2013)
High arginine content	Collagen formation, tissue repair, and wound healing	Tamrat et al. (2020)
Relatively high calcium	Healing fractured bones	Bizuneh (1984)
Zinc	Dietary nutrients	Heuzé et al. (2017); Tamrat et al. (2020)
Iron	Dietary nutrients	Heuzé et al. (2017); Tamrat et al. (2020)

As described in Section 3.2, the dominantly used part of enset is corm. The molecular and phytochemical profile of enset corm shows that it has a high amount of Calcium, Zinc, Iron, and Arginine contents, which have tremendous health benefits that are related to the traditional medicinal use of enset (Forsido et al. 2013; Tamrat et al. 2020). These findings correlate closely with traditional medicinal practices, particularly the dominant use of the corm in treating bone and tissue‐related conditions.

#### 3.5.1 High Arginine and Calcium Content of Enset Corm as Evidence for Bone‐Related Applications

Free amino acid profiling of enset landraces reveals that the corm is rich in arginine, which is vital for collagen synthesis, tissue regeneration, and wound healing (Tamrat et al. 2020). Arginine can stimulate collagen production both directly (via proline synthesis) and indirectly (as a precursor of nitric oxide), providing a biochemical explanation for the traditional use of *enset* in healing fractures and repairing body tissues (Tamrat et al. 2020). In addition, nutritional studies of enset demonstrated that enset products contain higher calcium concentrations than most cereals, tubers, and root crops (Bizuneh 1984). Given calcium's critical role in bone mineralization and repair, this further supports the longstanding practice of using *enset* for treating bone fractures and joint displacements.

#### 3.5.2 Antioxidants, Antitumor, Antibacterial, Nematicidal, and Antifungal Activity of Enset

A bioactive compound with antibacterial and antifungal properties has been isolated from enset (Hölscher and Schneider 1998), providing a scientific basis for its traditional medicinal use of enset by society. This study isolates a compound phenylphenalenone, which has demonstrated antitumor, antibacterial, nematicidal, and antifungal activity, highlighting the therapeutic potential of enset. Complementary studies further support these findings. Forsido et al. (2013) reported that enset possesses a moderate total phenol content and antioxidant capacity, with “Amicho” (corm of enset) exhibiting the highest ferric reducing antioxidant power (FRAP), indicating strong antioxidant potential (Table 5). Polyphenolic antioxidants play a crucial role in the body's defense system by scavenging reactive oxygen species, which are the harmful byproducts generated during normal cell metabolism, thereby reducing oxidative stress and associated disease risks (Fereja et al. 2024; Gutteridge and Halliwell 2006; Goanar et al. 2024; Ismael et al. 2021; Kumaran and Karunakaran 2007; Ou et al. 2002).

**TABLE 5:** Mean total antioxidant capacity and total phenolic content of different parts/products of enset.

Flour types	FRAP	DPPH	Phenolic content
Amicho	1.52	10.70	26.8
Unfermented kocho	1.17	10.70	55.9
Fermented kocho	1.00	16.31	47.9
Unfermented bulla	0.43	10.50	30.4
Fermented bulla	0.90	13.95	53.5
Stem	0.07	1.36	7.8

Abbreviations: DPPH, Di‐phenyl Poly Phenyl Haydrazine; FRAP, ferric reducing antioxidant power.

*Source:* Forsido et al. (2013).

#### 3.5.3 Enset as a Potential Dietary Source of Essential Micronutrients

Beyond the specific therapeutic compounds discussed earlier, Enset also serves as a significant source of essential dietary micronutrients, which underpin its broader health benefits. This is particularly evident in its content of iron and zinc, two micronutrients of major public health concern in Ethiopia.

In a comparison with regionally important tuber and cereal crops, enset contains notably high concentrations of iron and zinc (Tamrat et al. 2020). Anemia, primarily caused by iron deficiency, affects 56% of children and 24% of adult women in the country (Grebmer et al. 2018), highlighting the importance of enset as a potential dietary source of iron. Iron concentrations have been reported as higher in the enset pseudostem compared to the corm (Heuzé et al. 2017; Tamrat et al. 2020). Similarly, zinc deficiency is a major dietary concern in Ethiopia (Gebru et al. 2018). Evidence shows that enset is an important source of zinc, with the corm containing higher concentrations than the pseudostem (Tamrat et al. 2020). These findings underscore the significant nutritional, as well as medicinal, value of *enset* in improving public health outcomes (Dilebo 2025b). The role of other key nutrients like calcium and arginine in enset's medicinal applications is discussed in the preceding sections.

#### 3.5.4 Genetic Distinctiveness of Medicinal Landraces

Molecular characterization studies have demonstrated the genetic diversity of enset landraces. Nuraga et al. (2022) analyzed 38 medicinal enset landraces collected from different enset growing areas of southern Ethiopia and found moderate genetic variability (He = 0.47). The majority of the variation was contributed by variation among individuals, indicating low genetic differentiation among the groups. Importantly, nearly all landraces with distinct vernacular names were genetically distinct, suggesting that vernacular classification is a reliable proxy for genetic distinctiveness. Tsegaye (2002) similarly reported that medicinal landraces often showed unique AFLP bands, pointing to the presence of genes potentially linked to medicinal properties. These findings highlight the importance of combining indigenous knowledge with molecular tools to conserve and utilize medicinal *enset* sources.

### 3.6 Challenges for Medicinal Enset Landraces

The conservation and sustainable use of medicinal *Enset* landraces faces multiple challenges (Mitiku et al. 2024):

- Biotic and abiotic stresses: Production is constrained by pests, diseases, and environmental stresses, leading to genotype losses (Getachew et al. 2014; Matheka et al. 2019).
- Neglected conservation efforts: Medicinal landraces underrepresented in collections and preservation programs (Dalle and Daba 2021).
- Socioeconomic dynamics: Medicinal plants are often distributed freely by farmers without economic incentive to propagate or replant these specific landraces, limiting their sustainability.
- Wildlife pressure: Medicinal landraces are highly attractive to wild animals such as porcupines and pigs, contributing to losses (Negash 2007).
- Low yields and susceptibility: In regions like the Gurage Zone, medicinal landraces often have lower yields and greater vulnerability to diseases and drought, posing additional challenges to their survival (Nuraga et al. 2019). Consequently, these landraces are typically cultivated by farmers with larger land holdings, further limiting their distribution and propagation.

These factors underscore the urgent need for community‐based conservation, propagation, and sustainable management strategies that address both the biological and socio‐economic factors threatening these invaluable genetic resources (Dilebo 2025a, 2025b). By prioritizing the sustainable management and conservation of medicinal enset landraces, we can safeguard their continued availability and contribute to the preservation of traditional medicinal knowledge for future generations.

### 3.7 Future Thrust

There are several promising avenues to explore regarding the traditional medicinal use of *Enset* in Ethiopia. Future research and conservation efforts should focus on the following areas:

- Ethnobotanical research: Comprehensive documentation of indigenous medicinal practices and knowledge related to *Enset*.
- Phytochemical and pharmacological studies: Isolation, characterization, and evaluation of bioactive compounds for therapeutic potential through in vitro and in vivo studies.
- Conservation strategies: Community‐driven conservation initiatives, agroforestry practices, and genetic resource preservation, particularly targeting medicinal landraces.
- Integration with healthcare: Collaboration between traditional healers and modern healthcare professionals to develop evidence‐based treatment guidelines.
- Commercialization opportunities: Development of value‐added *Enset* products for medicinal and nutritional applications to enhance economic benefits.
- Capacity building: Strengthening interdisciplinary collaboration through training, workshops, and outreach to ensure sustainable utilization and conservation of *Enset*.

By pursuing these future research directions, we can further unravel the potential of enset as a valuable resource for traditional medicine, contribute to the preservation of indigenous knowledge, and promote the sustainable development of enset‐growing regions in Ethiopia and beyond.

## 4 Conclusion

Enset ( *Ensete ventricosum* ) plays a central role in Ethiopian traditional medicine, with approximately 16.7% of over 600 documented landraces documented for medicinal purposes. The Gurage Zone and Amaro Special District host the highest number of enset medicinal landraces. Various enset parts and products, notably the corm and bulla, are widely used in treating diverse ailments. Scientific evidence, including phytochemical and nutritional studies, underscores enset's potential as a medicinal commodity. However, conservation challenges, including disease susceptibility, environmental stress, and socio‐economic practices, threaten the sustainability of medicinal landraces. Strengthening conservation, scientific validation, and sustainable utilization strategies are therefore essential to preserve enset's role as both a medical and nutritional resource for future generations.

## Author Contributions

**Mitiku Muanenda:** conceptualization (lead), data curation (equal), formal analysis (lead), funding acquisition (lead), investigation (lead), methodology (equal), project administration (lead), resources (lead), software (equal), validation (equal), visualization (equal), writing – original draft (lead), writing – review and editing (equal). **Workineh Mengesha Fereja:** conceptualization (equal), data curation (equal), formal analysis (equal), funding acquisition (equal), investigation (equal), methodology (equal), project administration (supporting), resources (equal), software (equal), validation (equal), visualization (equal), writing – original draft (equal), writing – review and editing (lead). **Wadzani Palnam Dauda:** conceptualization (equal), data curation (equal), investigation (supporting), methodology (supporting), software (supporting), supervision (lead), validation (equal), visualization (supporting), writing – original draft (supporting), writing – review and editing (equal).

## Ethics Statement

This study was conducted after it was ethically reviewed and approved by the Research and Ethical Review Committee of the Department of Biology, College of Natural and Computational Science, Dilla University.

## Consent

All the authors declare that they have read and approved the final version of the manuscript and agree that the work is ready for submission for publication.

## Conflicts of Interest

The authors have declared that there are no conflicts of interest.

## Data Availability

The datasets used and/or analyzed during the current study are included in the manuscript.
